# Intimate partner violence and depression among pregnant women in the North west region of Cameroon: a research proposal

**DOI:** 10.1186/s13104-018-3979-0

**Published:** 2018-12-06

**Authors:** Tsi Njim, Franklin Ngu Mbolingong

**Affiliations:** 1 Health and Human Development (2HD) Research Network, Douala, Cameroon; 2 Sub-divisional Hospital Mankon, Bamenda, Cameroon

**Keywords:** Intimate partner violence, Depression, Cameroon, Violence against women instrument, Patient Health Questionnaire-9

## Abstract

**Objectives:**

Intimate partner violence (IPV) in pregnancy is a major public health concern due to its harmful effects on both the mother and the unborn foetus. In this study, we aim to assess the prevalence and correlates of both IPV and depression in pregnant women in the northwest region of Cameroon. Specifically: (1) To determine the prevalence of IPV in a group of pregnant women in the northwest region of Cameroon. (2) To determine the prevalence of depression amongst these women. (3) To assess the various sociodemographic determinants of IPV in these women. (4) To determine if IPV is associated with depression and to assess other sociodemographic and clinical correlates of depression.

**Results:**

This cross-sectional study will include a minimum of 369 pregnant women recruited by convenience sampling from primary and secondary healthcare facilities in the northwest region of the country. Data be collected via a printed questionnaire administered by a trained healthcare professional. IPV will be assessed using the World Health Organisation Violence Against Women Instrument and depression will be assessed using the Patient Health Questionnaire-9. Multivariable logistic regression will be used to identify independent predictors of IPV and depression.

## Introduction

Intimate partner violence (IPV) as defined by the World Health Organisation (WHO) is “any behavior within an intimate relationship that causes physical, sexual or psychological harm, including acts of physical aggression, sexual coercion, psychological abuse and controlling behaviors” [1, 2]. It is the commonest form of violence against women [3] and has become a huge public health issue [2, 3] especially in sub-Saharan African countries where the prevalence of IPV against women could be as high as 61% as shown in a cross-sectional analysis in northwestern Tanzania [4]. In Cameroon, it has been estimated from Demographic Health Survey (DHS) data in 2004 that approximately 38.7% of women experienced physical violence; 30.7% experienced emotional violence; and 14.8% experienced sexual violence from their intimate partners throughout their lifetime. IPV is also a serious problem during pregnancy. The proportion of women who have experienced IPV in pregnancy in sub-Saharan Africa ranges from 7 to 20% for physical violence [5–8]; 9.7–18% for sexual violence [6–8]; and 17–29% for psychological violence [6, 7].

IPV is frequently associated with negative consequences for women. These could include physical injuries like bruises, abrasions, lacerations, broken bones and/or teeth and, attempted strangulation [9, 10]. These women are also more likely to suffer from mental health problems like anxiety and phobias which could lead to some behavioural changes including alcohol and recreational drug abuse; smoking; poor self-esteem; post-traumatic stress disorder (PTSD) and unsafe sexual behavioural patterns [9, 10]. IPV is consistently associated with depression which is a leading cause of disability worldwide [2, 3, 11–13]; due to its debilitating symptoms which sometimes culminate in suicide [14].

Due to the sexual violence component of IPV, some of these women end up with unintended pregnancies which may end up in unsafe abortions especially in resource-limited settings; sexually transmitted infections including HIV and the sequelae of pelvic inflammatory diseases [7]. The association of IPV and spontaneous abortions and preterm deliveries could lead to the negative consequences associated with premature infants like respiratory distress and neonatal death [7]. Literature also explains that the effects of IPV on the infant extend to psychological issues like problems with attachment, behavioral and social disorders [15].

In Cameroon, there is a huge need for adequate mental health services as most healthcare providers lack adequate knowledge concerning mental health issues [16]. To fill this gap in knowledge, data needs to be made available on the prevalence of some mental health disorders especially in vulnerable populations. This will be the first study which determines the prevalence of IPV and depression in pregnancy in Cameroon and the relationship between the two constructs.

## Main text

### General objective

To determine if IPV is associated with depression in a group of pregnant women in the northwest region of Cameroon.

### Specific objectives

1. To determine the prevalence of IPV (physical, sexual and psychological violence) in a group of pregnant women in the northwest region of Cameroon.
2. To determine the prevalence of depression amongst these women.
3. To assess the various sociodemographic determinants of IPV in pregnancy in these women.
4. To determine if IPV is associated with depression in these women and to assess other sociodemographic and clinical correlates of depression in these women.

### Methods

#### Population and design

##### Population, sampling and sample size

The sampling frame includes all pregnant women in the northwest region of the country during the time of the study. Pregnant women reporting for antenatal consultations (ANC) and delivery in hospitals in this region of the country will be recruited by a consecutive sampling until a minimum sample size is attained, which is calculated by the formula below [17]: $n = \frac{{Z^{2} P \left( {1 - P} \right)}}{{d^{2} }}$

Assuming that the prevalence of IPV in our setting is 39.8% (using Demographic and Health Surveys estimates from Cameroon amongst women aged 20–24 years) [18]; and n = sample size, Z = Z statistic for a level of confidence, P = expected prevalence of IPV in pregnant women (39.8%, P = 0.398), and d = precision (in proportion of one; if 5%, d = 0.05). Z statistic (Z): For the level of confidence of 95%, which is conventional, Z value is 1.96.

A minimum of 369 pregnant women will be required for the study.

##### Design

This will be a cross-sectional study that will run for a period of 3 months. Pregnant women seeking ANC or delivery services at primary (The Mankon Medicalised Health Centre, Nkwen Medicalised Health Centre, District Hospital Santa) and secondary healthcare (The Regional Hospital Bamenda) facilities in the northwest region of the country will be recruited by a consecutive sampling. The Regional Hospital Bamenda is a second level hospital in the North west region which acts like a referral hospital in the region. It has approximately 300 outpatient consultations a day. Recruitment will be done in a consecutive manner in all four health care facilities until the sample size is attained.

The antenatal care consultation process is initiated by the nurses-in-charge and concludes with a visit to a doctor in high-risk patients. The nurses-in-charge will inform the patients of the study in its entirety. They will be informed that they have the choice to decline in participating in the study at any time they so wish. If the woman agrees to take part in the study, they will be directed to see the medical doctors who are the only researchers administering the questionnaires. The doctors will also explain the study to the participants again and seek informed consent from the participants. Due to the sensitive nature of the topics discussed, the questionnaires will be administered by medical doctors only with experience in psychiatry clinical rotations in a safe and confidential location within the health services. These doctors will receive a 1-week refresher course on IPV from a psychiatrist prior to data collection. Participants who are determined to exhibit severe depressive symptoms will be referred on to a psychiatrist at the Regional Hospital Buea and Presbyterian Hospital Douala who has been informed of the study; while those with moderate depressive symptoms will be referred to the psychosocial services in these hospitals.

##### Instrument

Data will be collected using a printed structured questionnaire divided into three sections where the following characteristics will be collected:

Section A: socio-demographic characteristics (age, marital status, educational status, average monthly income, job satisfaction, alcohol and recreational drug use by participant and partner, partner’s age, partner’s monthly income, partner’s level of education and number of sexual partners); clinical characteristics (gravid status, gestational age, age at first coitus and presence of any chronic medical illnesses). These clinical characteristics will be extracted from medical records. Where such records do not exist, the characteristics will be self-reported.

Section B: This section will use an adaptation of the WHO Violence against women instrument (VAWI) to collect information pertaining to IPV in pregnancy. This instrument has four questions on psychological violence, six questions on physical violence and three questions on sexual violence answered in Likert-scale format with responses ranging from “No” to “Yes—once, a few or many times”. This instrument has been shown to be valid and has a high internal consistency to discriminate against the various forms of IPV against pregnant women [19].

Section C: Screening for depression will be assessed using the nine-item Patient Health Questionnaire (PHQ-9) which has been shown to have a high sensitivity and specificity for the diagnosis of major depression [20].

### Data management and statistical analysis

Data entry will be done using EPI Info version 7 (CDC, Atlanta) and a random sample of 10% of data will be crosschecked to ensure accurate entry. Dummy analysis will be performed regularly to also ensure correct data collection. Data analysis will be done using Stata/IC version 15.0 Texas, USA.

#### Objective one

*To determine the prevalence of IPV (physical, sexual and psychological violence) in a group of pregnant women in the northwest region of Cameroon.*

The number of women who respond “yes” to at least one of the questions in each of the subsections of the VAWI (physical violence, sexual violence and psychological violence) will be assessed as a ratio against the total number of participants, to obtain the prevalence of IPV for each sub-category of the construct.

#### Objective two

*To determine the prevalence of depression amongst these women.*

A provisional diagnosis of depression will be made if PHQ-9 scores are greater than 4. The total number of women with scores above 4 will be used to calculate the prevalence of depression in the study population after obtaining a ratio of this number on the total number of participants. The severity of depression will be determined using the following classification: mild: 5–9; moderate: 10–14; moderately severe: 15–19; and severe: 20–27 [20].

#### Objective three

*To assess the various determinants of IPV in pregnancy in these women.*

A univariable analysis will be carried out to assess potential determinants of each subcategory of IPV using the Chi squared test for comparisons. Significant variables (p value < 0.05) will be inputted into a multivariable logistic regression model to obtain independent predictors for IPV.

#### Objective four

*To determine if IPV is associated with depression in these women and to assess other sociodemographic and clinical correlates of depression in these women.*

A univariable analysis using subcategories of IPV (physical, psychological and sexual violence) as predictors and depression (PHQ-9 scores > 4 and PHQ-9 scores ≤ 4) as the outcome using the Chi squared test for comparisons will be performed. Other sociodemographic and clinical variables will also be compared across categories of the outcome to obtain potential predictors of depression in these women. Significant variables will be inputted into a multivariable logistic regression model to obtain independent predictors of depression.

### Discussion

In this study, we will attempt to determine the prevalence and determinants of IPV and depression in pregnant women in the northwest region of Cameroon. IPV is increasingly being recognized as a significant public health concern around the world and its association with mental health conditions indicates the burden and morbidity associated with this type of violence; especially as these mental health conditions like depression are highly neglected in developing countries and in Cameroon in particular [21, 22].

Women who experience physical, sexual and psychological violence during pregnancy are more prone to suffer from debilitating mental health disorders including sleep disorders, anxiety and depression [7, 23]. This is particularly worrisome as the association of this condition and other foetal complications like spontaneous abortions is well established [7, 24, 25]. The impact of IPV on maternal and child health can therefore not be underestimated.

Being the first study of its kind in the country, the data obtained from this study will help to generate a hypothesis on the relationship between IPV and depression in this special population and add to the growing cartography of mental health issues and their correlates in Cameroon. This will help inform policy makers on the need for early detection of predictors of IPV in pregnant women and the investigation of preventive measures to reduce the probable progression to depression; and curb its burden on maternal and child health.

## Limitations

To the best of our knowledge, this is the first study which assess the prevalence and correlates of IPV and depression in pregnant women in Cameroon. Due to the cross-sectional nature of the study, temporal associations and causality between the outcomes and established predictors will be difficult to infer. Some of the clinical characteristics will be self-reported; this could lead to a potential recall bias.

## Abbreviations

IPV: intimate partner violence
WHO: World Health Organisation
VAWI: violence against women instrument
ANC: antenatal care
PHQ-9: Patient Health Questionnaire
